# Sustainable Synthesis of Zinc Oxide and Its Application in Water Contaminant Removal

**DOI:** 10.3390/nano16191244

**Published:** 2026-10-01

**Authors:** Piotr Dulian, Olena Sachuk, Olena Kiziun, Liudmyla Kotynska, Valery Zazhigalov, Marta Żurek, Janusz Jaglarz, Witold Żukowski

**Affiliations:** 1 Faculty of Chemical Engineering and Technology, Cracow University of Technology, 24, Warszawska Str., 31-155 Cracow, Poland; 2 Interdisciplinary Center for Circular Economy, Cracow University of Technology, 24, Warszawska Str., 31-155 Cracow, Poland; 3 The State Scientific Research Forensic Center of the Ministry of Internal Affairs of Ukraine, 4, Velyka Okruzhna Str., 03680 Kyiv, Ukraine; 4 Institute for Sorption and Problems of Endoecology, National Academy of Sciences of Ukraine, 13, Oleg Mudrak Str., 03164 Kyiv, Ukraine; 5 Doctoral School, Cracow University of Technology, 24, Warszawska Str., 31-155 Cracow, Poland; 6 Faculty of Materials Engineering and Physics, Cracow University of Technology, 37, JPII St., 31-155 Cracow, Poland

**Keywords:** photocatalysis, zinc oxide, circular economy, sustainable synthesis, wastewater treatment

## Abstract

This study explores the sustainable synthesis of zinc oxide (ZnO) from industrial waste and its application in removing water contaminants such as chloramphenicol (CAP), metronidazole (MNZ), and prometryn (PROM). ZnO synthesized from zinc production waste (ZnO(2)) exhibited competitive photocatalytic performance compared to commercial ZnO (ZnO(1)), achieving 79% CAP degradation (Kd = 0.53 × 10^−2^ min^−1^) and 72% MNZ degradation (Kd = 0.43 × 10^−2^ min^−1^) under UV light, while showing higher activity in PROM degradation (32% removal) due to its mesoporous structure. Although ZnO(2) had a slightly lower specific surface area (24.9 m^2^/g) and pore volume (0.26 cm^3^/g) than ZnO(1), it demonstrated significant environmental and economic benefits by repurposing industrial waste. Catalytic tests in ethanol oxidation revealed ZnO(2) required higher temperatures for full conversion but exhibited higher acetaldehyde selectivity (70%). These findings highlight the potential of waste-derived ZnO as a sustainable alternative for water treatment and catalytic processes, aligning with circular economy principles and reducing reliance on natural resources.

## 1. Introduction

The development of the agrochemical and pharmaceutical industries generates significant environmental challenges, especially due to the presence of persistent contaminants such as herbicides and pharmaceuticals in surface and groundwater. These compounds exhibit high durability and resistance to biodegradation, leading to their accumulation in the environment and posing toxicological risks to aquatic organisms and humans. Most of these contaminants are not efficiently removed by conventional municipal wastewater treatment plants, which results in their continuous release into natural water resources [1,2]. Furthermore, some of these substances can undergo transformation processes in the natural environment, forming derivatives that often exhibit even higher toxicity to living organisms [3,4]. For instance, in works [5,6] it was shown that the photolysis of AB chloramphenicol (CAP) in aqueous solution under UV and visible lights leads to the formation of a number of toxic substances, namely hydrochloric acid, p-nitrobenzaldehyde, p-nitrobenzoic acid, 4,4 azoxybenzoic acid, and p-aminophenyl-2-acetamido-l,3-propanediol. In turn the negative impact of metronidazole (MNZ) on the human body is well documented [7,8]. It was reported that the maximum concentration of MNZ was at 9400 ng/L and 127 ng/L, respectively, in hospital wastewater [9] and the wastewater treatment plants [10].

Photocatalytic processes involving semiconducting metal oxides such as ZnO and TiO_2_ currently represent some of the most advanced methods for the effective deactivation of organic contaminants [11]. A key advantage of photocatalysis over conventional filtration and sorption technologies is its ability to achieve complete mineralization of pollutants, resulting in their permanent decomposition into simple, non-toxic inorganic substances instead of merely facilitating their transfer between phases. Among the wide range of photocatalytic materials, zinc oxide is of particular interest as it combines a wide band gap and high charge carrier mobility with high availability and a lower production cost compared to other popular photocatalysts, such as TiO_2_ or semiconductors containing noble metals. Owing to these properties, ZnO remains one of the key materials studied for both laboratory and environmental applications [12].

In the context of circular economy principles, the development of ZnO-based photocatalysts from waste streams has recently attracted substantial scientific attention. Recent research increasingly focuses on utilizing various types of industrial, agricultural, and metallurgical byproducts as well as waste materials as alternative precursors for the synthesis of functional materials, including photocatalysts. This strategy not only allows for the recovery and conversion of valuable constituents present in these waste streams—reducing their environmental burden—but also enables the implementation of sustainable chemical processes by integrating secondary resources into closed-loop material cycles [13,14,15]. By employing waste-to-product strategies for ZnO synthesis, resource consumption can be minimized and production costs decreased. The latest scientific reports have demonstrated that ZnO derived from waste sources may reach photocatalytic performance comparable to that of conventional materials synthesized from high-purity commercial precursors, offering a pathway to more ecologically and economically viable applications of semiconductor photocatalysts [8,16,17,18,19,20,21].

In this article, an original method for obtaining a zinc oxide photocatalyst is presented, utilizing a real waste stream generated during industrial zinc production. A complete process for the conversion of electrolyte waste leading to the production of ZnO with large surface area and photocatalytic properties is described. Particular emphasis has been placed on determining the efficiency of degradation of selected persistent organic contaminants using the obtained material and on directly comparing its properties with reference zinc oxide from commercial sources. The results obtained not only enable assessment of the practical potential of waste-derived photocatalysts but also offer insight into the challenges and opportunities related to the implementation of circular economy principles in the production of advanced functional materials. The scientific objective of this work was to verify whether real waste streams from industrial zinc production can be used to obtain ZnO with photocatalytic effectiveness comparable to commercial materials. Although ZnO recovery from zinc-containing secondary resources has been reported, studies addressing the conversion of a real post-production acidic zinc electrolyte into ZnO and its direct comparison with commercial ZnO in photocatalytic and catalytic applications remain limited [16].

## 2. Materials and Methods

The starting material was a genuine post-production acidic electrolyte collected from the hydrometallurgical zinc production line at ZGH Bolesław S.A. (Bukowno, Poland). The stream contained zinc as the main recoverable metal component, together with manganese and iron impurities originating from the industrial process. The developed method focuses on precipitating solid zinc salt from a waste solution of sulfuric acid, representative of secondary streams generated in non-ferrous metallurgy plants. This approach demonstrates the applicability of the proposed methodology to a real zinc-bearing industrial electrolyte and provides a basis for evaluating its transferability to related zinc-containing secondary streams. The synthesis procedure applied in this work is an original method developed by the authors specifically for the conversion of industrial zinc-bearing waste electrolyte into ZnO nanopowder; it has not been adapted from a previously published protocol (Table 1). The synthesis technique consisted of four distinct stages designed to remove impurities (particularly Mn and Fe) and convert zinc salts to high-purity ZnO nanopowder. In the first stage, zinc oxalate precipitation was performed by adding oxalic acid dihydrate to the waste sulfuric acid solution under controlled pH conditions. During this step, both zinc and manganese formed their respective oxalate compounds (Equations (R1) and (R2)). The second stage involved ammonization of the precipitated material using ammonium hydroxide solution, which resulted in the formation of tetraammine zinc oxalate complex (Equation (R3)) while simultaneously converting manganese compounds to manganese oxohydroxide (Equations (R4) and (R5)). The selective nature of this complexation allowed effective separation of manganese impurities through filtration. The third stage comprised deammonization and neutralization of the zinc ammine complex using dilute sulfuric acid, which regenerated zinc oxalate hydrate in purified form (Equation (R6)). Excess ammonium compounds were neutralized and removed as ammonium sulfate solutions (Equations (R7) and (R8)). The final stage involved thermal treatment of the purified zinc oxalate: initial drying at 110 °C for 2 h to remove crystal water (Equation (R9)) in a laboratory dryer, followed by calcination to decompose the oxalate and form crystalline zinc oxide nanopowder (Equation (R10)). Calcination was carried out in a laboratory muffle furnace (Nabertherm, Lilienthal, Germany) in air atmosphere at 450 °C for 4 h, with a controlled heating rate of 5 °C/min to ensure complete decomposition of the oxalate precursor while maintaining nanometric particle size. All experiments were conducted at laboratory scale, with individual batches yielding up to approximately 200 g of ZnO nanopowder.

**Figure 1:**
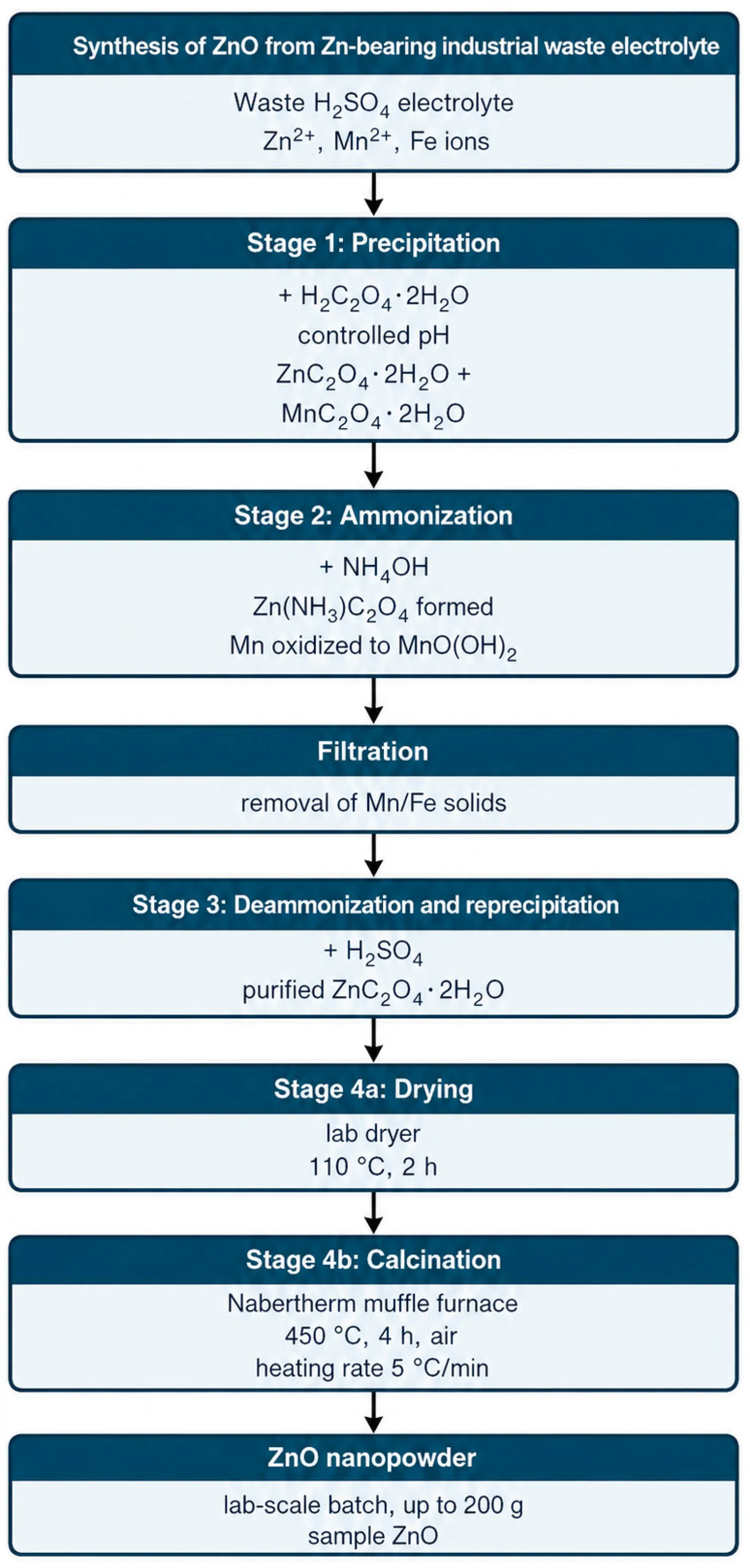
Schematic representation of the four-stage process converting Zn-bearing industrial waste electrolyte into ZnO nanopowder, including impurity removal (Mn and Fe) and thermal decomposition steps.

Throughout the synthesis, process parameters were optimized to maximize zinc recovery (>95%) while minimizing impurity content. The pH during precipitation was maintained at 2.5–3.0 to ensure selective oxalate formation. The ammonization step was conducted at pH 9–10 with a 4:1 molar ratio of ammonia to zinc. Temperature control during calcination was critical for preventing particle agglomeration while ensuring complete conversion to the oxide phase.

The specific surface area of each sample was determined by nitrogen physical adsorption at 77 K, using a Quantachrome NOVA-2200e analyzer (Boynton Beach, FL, USA). The surface areas were evaluated based on the standard BET procedure. The pore size distribution curves of the samples were calculated from desorption branches of the nitrogen isotherms by the BJH method.

Photocatalytic properties of zinc oxide powders (ZnO(1) commercial product—Umicore (CAS No. 1314-13-2) and ZnO(2) product made from liquid waste from zinc production) were studied in the oxidation of pharmaceuticals chloramphenicol (Levomycetin) and metronidazole, which are used as antibiotics, and herbicide Gezagard, where prometryn (PROM) is an active substance. The degradation experiments were conducted in two photoreactors equipped with a UV lamp (Optima, Andheri East, Mumbai, India, 125 W) and visible lamp bulb (Philips, Haryana, India, 70 W power) as irradiation sources.

An X-ray diffraction method (XRD) was applied to examine the crystalline structure of obtained ZnO powder. XRD diffractograms were recorded using a Philips X’Pert Prof diffractometer (CuKα radiation, λ = 1.5406 Å) in the 2θ range from 10° to 90°, with a step size of 0.01° (Philips). Qualitative identification of crystalline phases was performed based on the agreement of the obtained diffraction data with the PDF4+ cards.

The surface morphology and crystal sizes were examined using Scanning Electron Microscopy Apreo 2 S LoVac (Thermo Fisher Scientific, Waltham, MA, USA) and high-resolution, transmission electron microscopy HR-TEM TALOS F200X (Thermo Fisher Scientific, Waltham, MA, USA).

Chemical composition analysis was performed by inductively coupled plasma mass spectrometry (ICP-MS) using a Perkin Elmer ELAN 6100 instrument (PerkinElmer, Thane, India). Chlorine content was determined with limited sensitivity due to isobaric interferences typical for ICP-MS analysis of halogens. The analysis was performed as a single determination of the analyzed sample.

For the photocatalytic degradation studies, each solution (300 mL) with a certain concentration (Table 2) containing an appropriate dosage of ZnO powder (150 mg) was prepared in water in darkness. The suspensions were continuously magnetically stirred for 1 h to reach the adsorption equilibration between photocatalyst and organic pollutant in the dark before irradiation. Afterwards, aliquots (5 mL) of the solutions after irradiations were collected at 1 h intervals up to 5 h. The photocatalytic activities (constant degradation rate) of zinc oxide powders were estimated by measuring of optical density (absorbance) of CAP, MNZ and PROM solutions at appropriate wavenumbers by the UV–Vis spectrometer Shimadzu UV-2450 (Shimadzu Corporation, Kyoto, Japan).

The degree of photocatalytic degradation (G) was calculated from the relative decrease in pollutant concentration, assuming the Beer–Lambert relationship between concentration and absorbance, as commonly applied in photocatalytic degradation studies [6,22,23] using Equation (1):

(1) $$G=\frac{C_{0}-C}{C_{0}}\times 100=\frac{A_{0}-A}{A_{0}}\times 100$$

where *C*_0_ is the initial concentration of the substrate, *C* is the concentration of the substrate at a specified point in time, *A*_0_ is the initial solution absorbance (when t = 0 min), and *A* is the pollutant absorbance after 5 h of photoirradiation determined at λ_max_ of each pollutant.

CAP transformation products were analyzed by MKh-7304A monopole mass spectrometer (Electron, Sumy, Ukraine) with electron impact ionization, adapted for thermodesorption measurements.

Catalytic properties of samples were studied also in the selective ethanol oxidation reaction in the temperature range 110–410 °C. The parameters of catalytic activity of the samples were calculated according to the following Formulas (2)–(5):

*X_EtOH_* = *C_EtOH_*(*in*) − *C_EtOH_*(*out*)/*C_EtOH_*(*in*) × 100% (2)

*S* = *C*/*C_EtOH_*(*in*) − *C_EtOH_*(*out*) × 100% (3)

*Y* = *X_EtOH_* × *S*/100% (4)

(5) $$N=k\frac{Y_{\mathrm{Ac}}}{m_{\mathrm{cat}}}$$

where *X_EtOH_*—ethanol conversion %, *S*—selectivity of product %, *Y*—product yield %, *C_EtOH_*(*in*)—initial ethanol molar concentration, *C_EtOH_*(*out*)—ethanol molar concentration after reactor, *C*—product molar concentration after reactor, *N*—catalyst productivity for acetic aldehyde, mol/kg_cat_·h, and *k* (coefficient taking into account the amount of reagent passing through in 1 h) is 1.9 × 10^−5^ mol/h.

## 3. Results

### 3.1. Structure and Microstructure of the Materials

**Figure 2:**
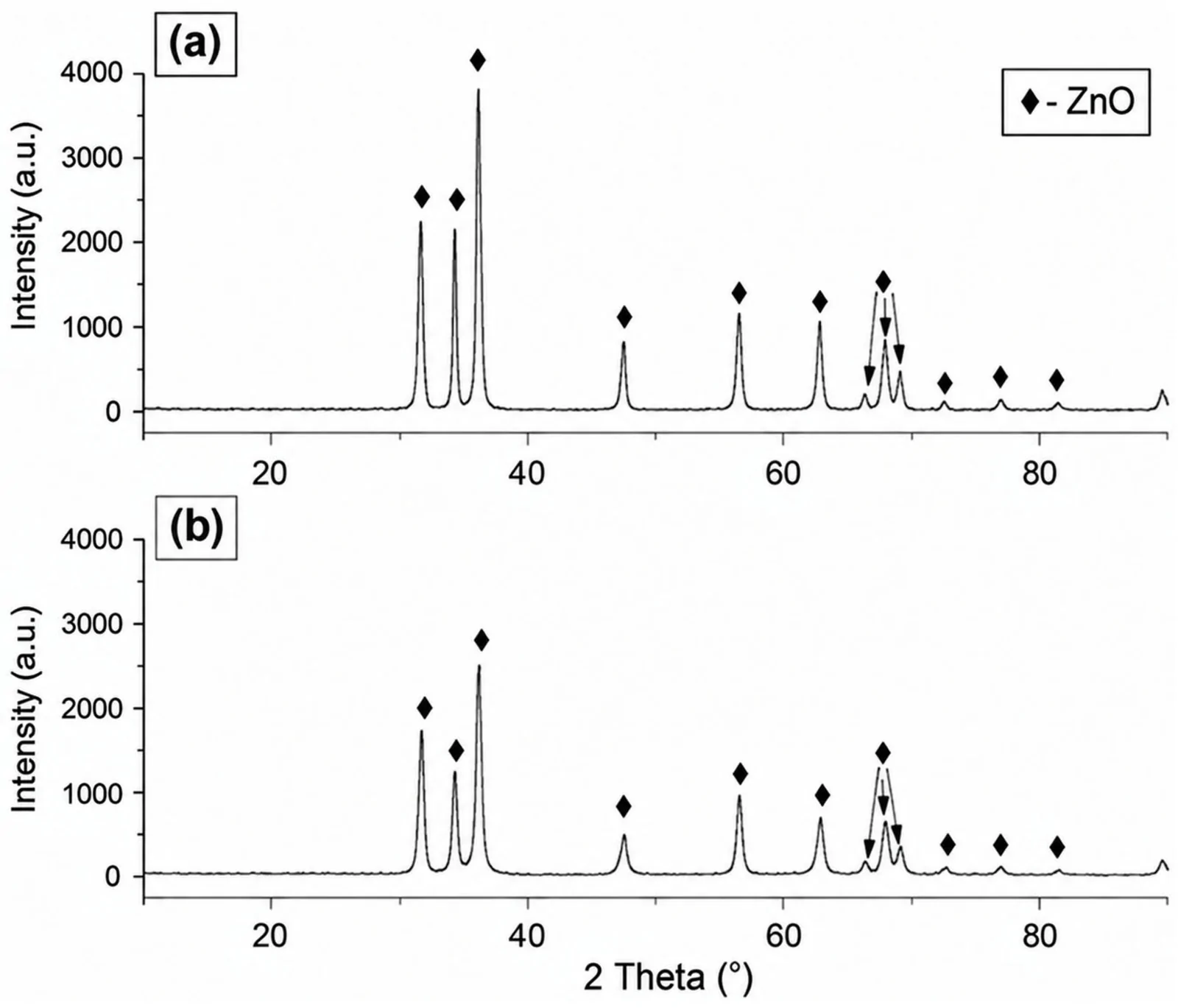
X-ray diffractograms for (**a**) the commercial ZnO and (**b**) the synthesized powder.

The crystal structure and phase composition of the obtained material (Table 1) were examined using the powder X-ray diffraction (XRD) method. Figure 2 presents a comparison of X-ray diffractograms for the synthesized powder (Figure 2a) and the diffractogram of the reference material used in this study, i.e., commercial ZnO (Figure 2b). All recorded diffraction peaks corresponded to the crystalline phase of zinc oxide. There were no peaks of other crystalline phases, confirming that the material is monophasic. This confirms the successful synthesis of the compound. All diffraction peaks were assigned to hexagonal wurtzite ZnO in agreement with the reference pattern (PDF No. 36-1451). The relatively intense reflection observed at 2*θ* ≈ 36° was assigned to the (101) planes, which constitute one of the characteristic strong reflections of powder wurtzite ZnO. A comparison of the intensity ratio *I*_(101)_/*I*_(100)_ for the synthesized sample and the reference pattern gave values of 1.47 and 1.50, respectively. Therefore, no significant preferred orientation was inferred for the waste-derived ZnO, and the observed diffraction pattern is consistent with a polycrystalline wurtzite material. The calculated crystallite sizes based on the analysis of the recorded X-ray diffraction data using the Debye–Scherrer method are very similar for both tested materials, approximately 40 nm.

**Figure 3:**
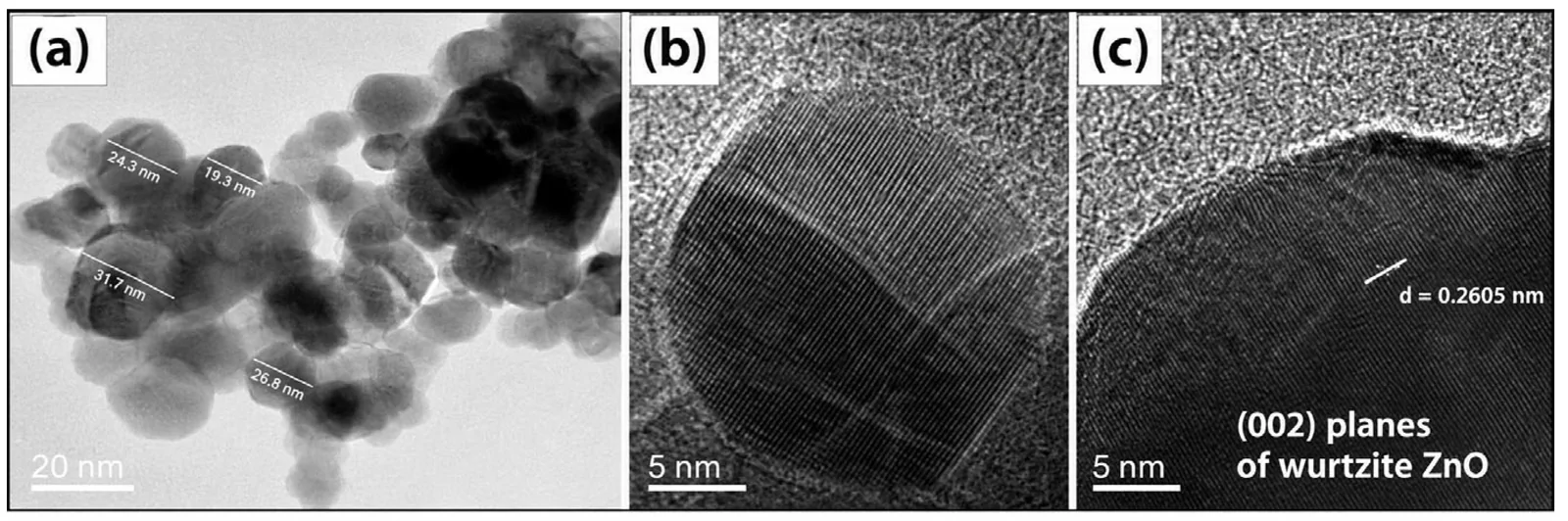
TEM and HRTEM characterization of waste-derived ZnO: (**a**) TEM micrograph showing agglomerated nanocrystals with representative particle dimensions in the range of approximately 18–39 nm; (**b**) lattice-resolved HRTEM image showing well-defined lattice fringes; (**c**) enlarged HRTEM image used for the measurement of an interplanar spacing of *d*_002_ = 0.2605 nm, assigned to the (002) planes of hexagonal wurtzite ZnO.

Representative TEM micrographs revealed aggregated nanocrystals with characteristic dimensions ranging from approximately 18 to 39 nm. Lattice-resolved HRTEM images (Figure 3b,c) clearly showed continuous and well-defined lattice fringes, confirming the high crystallinity of the waste-derived ZnO. The measured interplanar spacing of 0.2605 nm was assigned to the (002) planes of hexagonal wurtzite ZnO. This local structural identification is consistent with the XRD results, which confirmed the formation of phase-pure wurtzite ZnO (PDF No. 36-1451). The dimensions of individual nanocrystals observed by TEM are comparable in order of magnitude with the average crystallite size of approximately 40 nm estimated from XRD line broadening.

**Figure 4:**
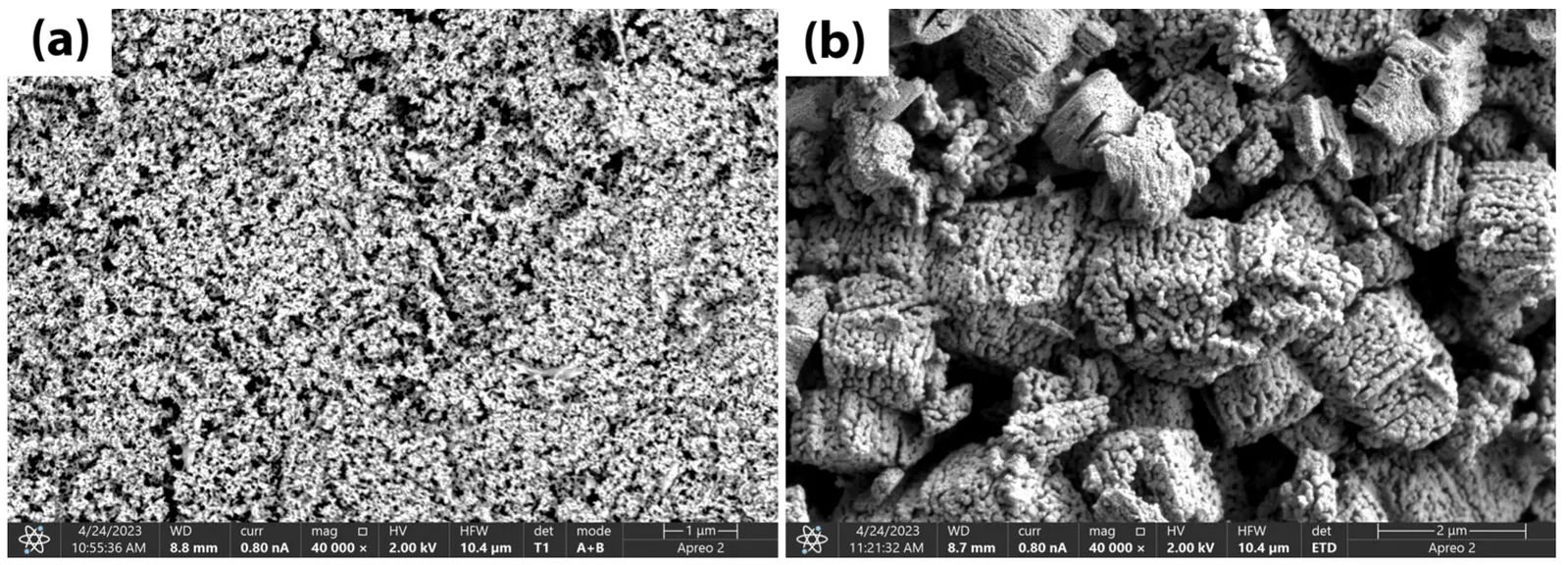
SEM micrographs of morphology of zinc oxide (**a**) from Umicore (**b**) synthesized by an alternative method.

Despite the significant similarities in crystallite size and crystal structure between these materials, surface morphology studies conducted using scanning electron microscopy (SEM) revealed substantial differences in grain morphology between the two materials. SEM images of the tested samples are presented in Figure 4. The reference sample exhibited a highly uniform distribution of grain sizes, with virtually no observed agglomerates. In contrast, zinc oxide synthesized from waste materials is characterized by a high degree of grain agglomeration. However, these agglomerates form loose structures with high porosity and a rough surface, making this material interesting for potential applications in catalytic processes. The observed morphology of the waste-derived ZnO can be related to the thermal decomposition pathway of the zinc oxalate precursor. During drying, removal of crystallization water from ZnC_2_O_4_·2H_2_O occurs, whereas, during calcination, the anhydrous oxalate is converted into ZnO with the evolution of CO_2_ (Table 1, Equations (R9) and (R10)). The release of gaseous products from precursor agglomerates may generate transient voids and inhibit complete densification of the particles. Together with the applied controlled heating rate, this process may contribute to the formation of the loosely packed, rough agglomerates observed by SEM and to the mesoporous structure identified by nitrogen adsorption–desorption measurements. This interpretation is consistent with the type-IV isotherm and the pore-size-distribution maximum at approximately 16 nm obtained for ZnO(2).

The elemental analysis of the waste-derived ZnO (Table 3) confirmed a high zinc content and only trace amounts (0.01–0.02 wt%) of transition-metal (Fe and Mn) and heavy-metal (Pb) impurities. These impurity levels are comparable to those typically reported for commercial ZnO powders used in photocatalytic applications. Such low dopant concentrations may introduce a limited number of trap centres that can assist charge carrier separation without causing the detrimental recombination effects usually observed at higher doping levels.

**Figure 5:**
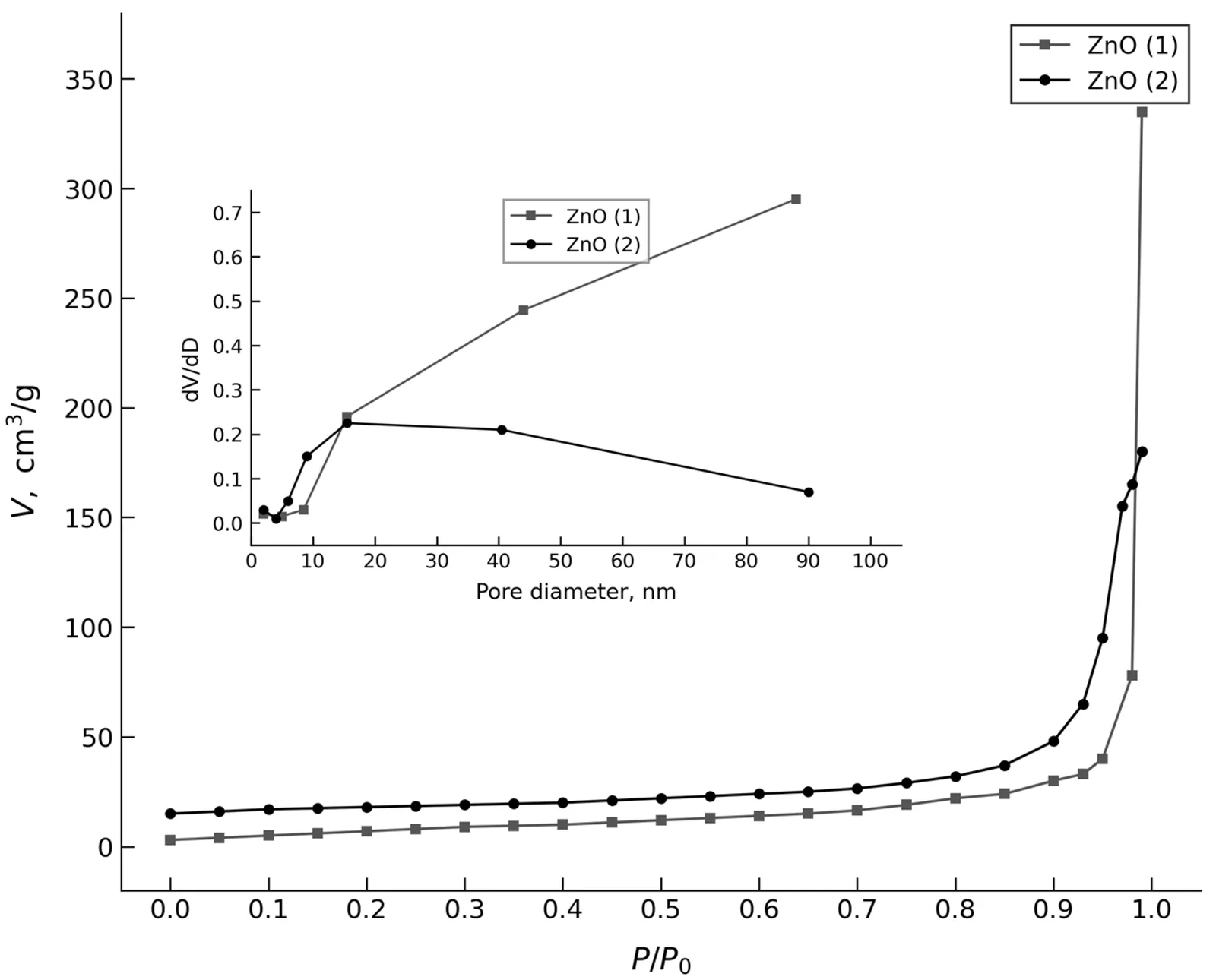
The nitrogen adsorption–desorption isotherms of the ZnO samples and the corresponding pore size distribution curves (inset).

The full nitrogen adsorption–desorption isotherms and pore size distribution curves (inset) of zinc oxides are shown in (Figure 5). We find that the type of adsorption–desorption isotherms and pore size for powders of zinc oxide are obviously different. As can be seen from Figure 5 (curve 1) the isotherm of ZnO(1) belongs to type II adsorption isotherms (according to IUPAC classification) with mono-polylayer adsorption [24]. Such a type of isotherms belongs to non-porous or macroporous adsorbents that are proved by the BJH method.

In contrast to the first sample, adsorption–desorption isotherms of ZnO(2) (Figure 5, curve 2) powder have an S-shaped character with a pronounced hysteresis loop in P/P_0_ region of 0.65–0.98. Curves of nitrogen sorption possess type IV indicating the presence of mesopores. According to the IUPAC classification, hysteresis loop has an H1 type [24]. This indicates the existence of wedge-shaped pores with open ends. However, with farther observation it can be seen that the hysteresis loop of ZnO(2) is small, indicating that its pore-size distribution is optimal. The mean diameter of pores can be gained from BJH pore size distribution plots (Figure 5 inset, curve 2). The presence of the maximum at 16 nm on the pore size distribution curve proves a formation of mesoporous structure in ZnO(2) sample. The values of specific surface area and total pore volume are presented in Table 4. The structural and textural differences between ZnO(1) and ZnO(2) are reflected in their substrate-dependent photocatalytic behaviour. ZnO(1), which exhibited a higher specific surface area (27.4 m^2^/g), a larger total pore volume (0.52 cm^3^/g), and a more uniform, less agglomerated morphology, showed higher photocatalytic efficiency toward chloramphenicol and metronidazole under UV irradiation. In contrast, ZnO(2) formed loose, rough, and mesoporous agglomerates and showed a higher degradation rate constant for prometryn, *K_d_* = 0.14 × 10^−2^ min^−1^, compared with 0.07 × 10^−2^ min^−1^ for ZnO(1). These results indicate that the photocatalytic response is not determined by the specific surface area alone. The porous aggregated structure of ZnO(2) may influence adsorption and mass transport processes at the catalyst solution interface, thereby contributing to its different behaviour toward prometryn. However, because dedicated adsorption isotherms and direct charge carrier measurements were not performed, this relationship should be regarded as a structure property interpretation rather than a direct mechanistic proof.

### 3.2. Photocatalytic Activity Tests

**Figure 6:**
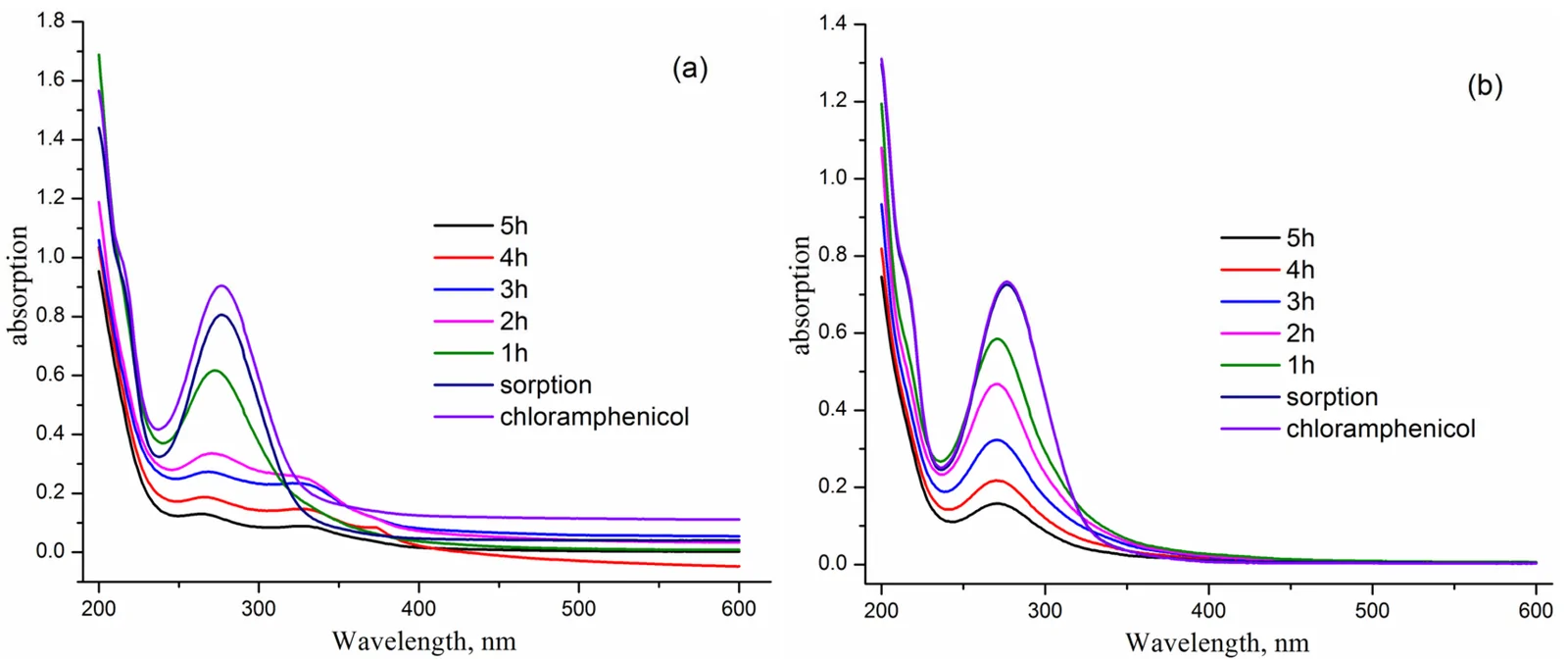
UV–vis absorption spectra of a CAP solution during 5 h of UV irradiation using ZnO(1)—(**a**) and ZnO(2) samples—(**b**).

The medicine drugs (CAP and MNZ) and herbicide (PROM) degradation experiments were conducted to evaluate the performance of zinc oxide photocatalysts under UV and visible irradiation. The UV–vis spectra of CAP solution with ZnO photocatalysts are presented in Figure 6. Obtained data show that both samples have a high activity in the degradation process of CAP solution. This is evidenced by electronic absorption spectra which are recorded for CAP (Figure 5) and results of constant rate of CAP degradation which are presented in Table 4. As can be seen from the results of aqueous CAP solutions in the presence of ZnO(1) (Figure 6a) and ZnO(2) (Figure 6b) photocatalysts, the irradiation under UV and visible (spectra are not given) lights leads to reducing intensity of the absorption maximum at 275 nm (the analogous tendency was observed in degradation process of MNZ and PROM solutions at appropriate λ_max_) and insignificant shifting of the main peak in the shortwave region (Figure 6). This behaviour can be attributed to changes in the structure of CAP molecule, namely electronic processes (see below).

It was established that the radiation is absorbed exclusively by the photocatalyst at the destruction of pollutant. The mechanism of formation of photocatalytic active surface of the ZnO particle includes the formation of holes in the valence band (h*_vb_*^+^) and electrons in the conduction band (e^−^*_cb_*) [25]. This process is initiated when the semiconductor absorbs photons with energy equal to or higher than its band-gap energy. Bulk ZnO is a wide bandgap semiconductor, with a reported band-gap energy of approximately 3.37 eV [26]. The holes contribute to the formation of hydroxide radicals *OH*^•^ and oxidation of organic compounds, while electrons lead to the formation of superoxide radicals $O_{2}^{•-}$ and decomposition reaction of organic molecule. The pair of electron–hole participates in redox reactions following two different mechanisms and can act as reducing/oxidizing agents for the degradation of species adsorbed on the photocatalysts or first react with surface adsorbed water molecules to produce *OH*^•^ according to Equations (6)–(9):

(6) $$\mathrm{ZnO}\overset{\mathrm{h\nu}}{\to }e^{-}+h^{+}$$

(7) $$\mathrm{ZnO}(h^{+})+H_{2}O/\mathrm{OH}\to \mathrm{ZnO}+{\mathrm{OH}}^{•}+H^{+}$$

(8) $$\mathrm{ZnO}(e^{-})+O_{2}\to \mathrm{ZnO}+O_{2}^{•-}$$

(9) $$h^{+},\;{\mathrm{OH}}^{•}+O_{2}^{•-}+\mathrm{organic}\;\mathrm{pollutant}\to \mathrm{oxidated}\;\mathrm{products}$$

Schematically, the mechanism of photoactivation of a semiconductor particle by ultraviolet radiation is presented in [27]. According to Equation (9) hydroxyl and superoxide radicals react with organic molecules with the oxidation of the latter to ions Cl^−^, CO_3_^2−^, NO_3_^−^, NH_4_^+^ and H_2_O. This fact was confirmed by data of TPDMS spectrometry (spectrum is not given). Obtained results peaks with mass numbers of 18, 19, 28 and 44, which correspond to H_2_O, CO and CO_2_, respectively, were detected. In fact, it can indirectly indicate the course of complete mineralization of chloramphenicol. Herewith, the values of the medium pH of CAP solutions during the photodegradation process were 5.4–6.3, which are in accordance with results in [27].

**Figure 7:**
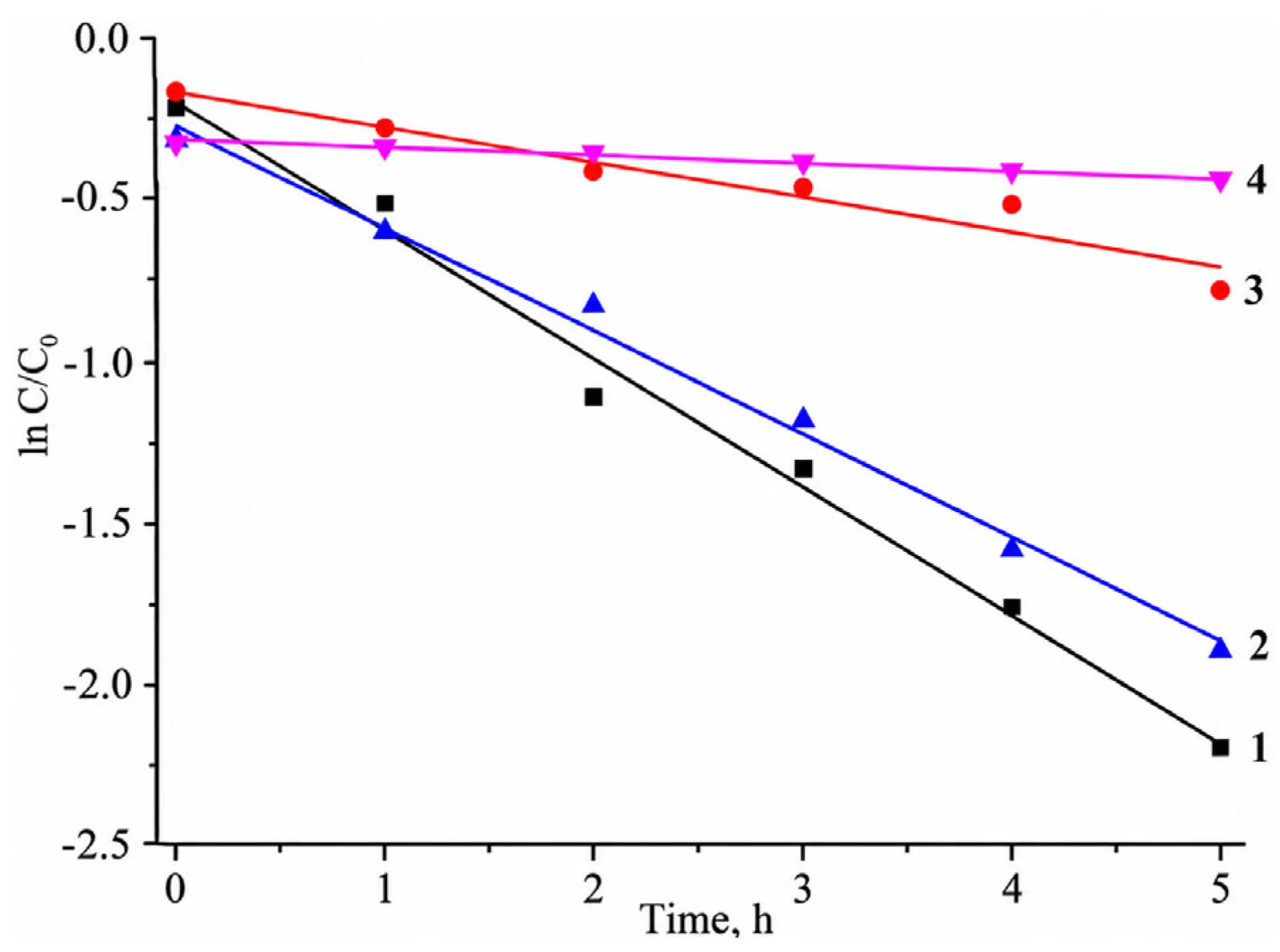
Kinetics of photocatalytic degradation of chloramphenicol solution in the presence of photocatalysts: ZnO(1) UV-1, ZnO(2) UV-2, ZnO(1) vis-3, ZnO(2) vis-4.

Kinetic curves of dependence lnC/C_0_-τ were constructed to compare the photocatalytic activity of the ZnO samples. An analysis of the experimental data indicates that the chloramphenicol is similar to the pseudo-first-order reaction (Figure 7) (a similar tendency was observed in photodegradation of metronidazole and prometryn) photodegradation process, i.e., kinetics of the change in the drug concentration can be described by Equation (10):

(10) $$\mathrm{kt}=\ln\frac{C_{0}}{C}$$

where *k* is the apparent reaction rate constant, *C*_0_ and *C* are the initial concentration and the reaction concentration of pollutant, respectively, and *t* is time.

As can be seen from Figure 7 ZnO(1) sample shows a higher photocatalytic activity compared with ZnO(2) powder. This fact can be explained by displacement of adsorption edge in the region of smaller wavelength and higher specific surface area which is available to drug molecules. The linear regressions showed good correlation coefficients: R = 0.995 and R = 0.966 for ZnO(1) under UV and visible irradiation, respectively, and R = 0.997 and R = 0.987 for ZnO(2) under UV and visible irradiation, respectively.

It is worth noting that the highest photocatalytic efficiency (degradation degree) on the ZnO powders is 86% observed for chloramphenicol oxidation under UV light, while the visible irradiation allows only 12–46% of efficiency to be achieved (Table 4).

**Figure 8:**
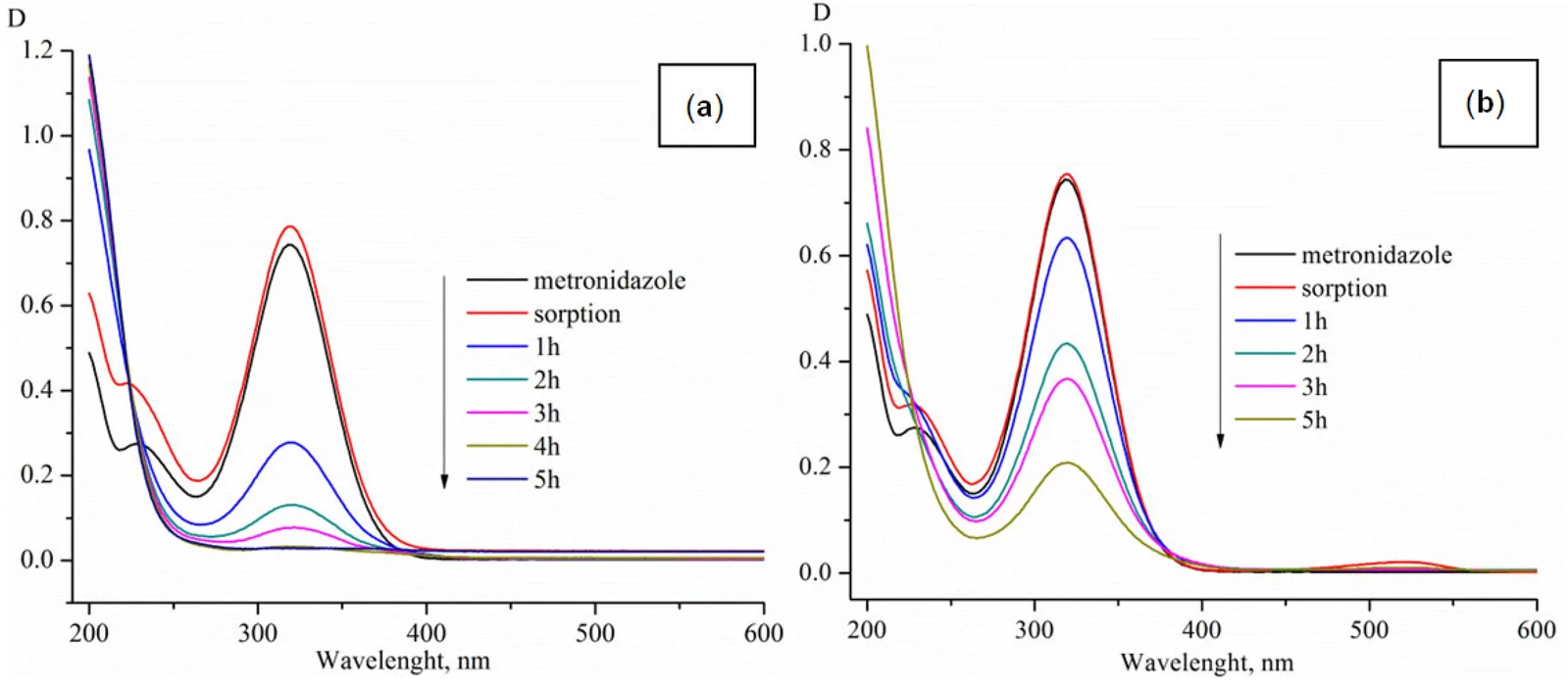
UV–vis absorption spectra of metronidazole solution during 5 h of UV irradiation using ZnO(1)—(**a**) and ZnO(2) samples—(**b**).

The spectra shown in (Figure 8) clearly illustrate the importance of photocatalysis in the elimination of MNZ. It was established that, after 5 h reaction time, the mineralization of MNZ is more effective on the ZnO(1) catalyst compared to ZnO(2). From Figure 8a it can be seen that, after 1 h of irradiation, the considerable reduction in the intensity of the absorption maximum at 320 nm occurs. The calculations of degradation rate constant and photocatalytic efficiency of MNZ (Table 4) prove the better photocatalytic properties of ZnO(1) sample. The possible pathways of MNZ photodegradation were suggested in [8,22]. Defect-related states, including oxygen vacancies, zinc interstitials, and surface-related centers, are known to influence charge trapping, charge-carrier recombination, and the formation of reactive oxygen species in ZnO photocatalysts [28,29]. In particular, such states can act as electron traps and may promote the activation of adsorbed oxygen, thereby facilitating the formation of superoxide and hydroxyl radicals involved in photocatalytic oxidation [30,31]. However, no direct defect-sensitive characterization, such as photoluminescence spectroscopy, electron paramagnetic resonance, or X-ray photoelectron spectroscopy, was performed in the present study. Therefore, the type and concentration of defect states in ZnO(1) and ZnO(2) cannot be determined or quantitatively compared. The differences in photocatalytic behavior are consequently discussed primarily in terms of the experimentally established surface area, pore structure, morphology, and crystallite dimensions. Direct characterization of defect-related states will be considered in future work. Given promising results obtained on ZnO samples in antibiotic destruction reactions simultaneously, the experiments for removal of herbicide from aqueous solution were performed.

**Figure 9:**
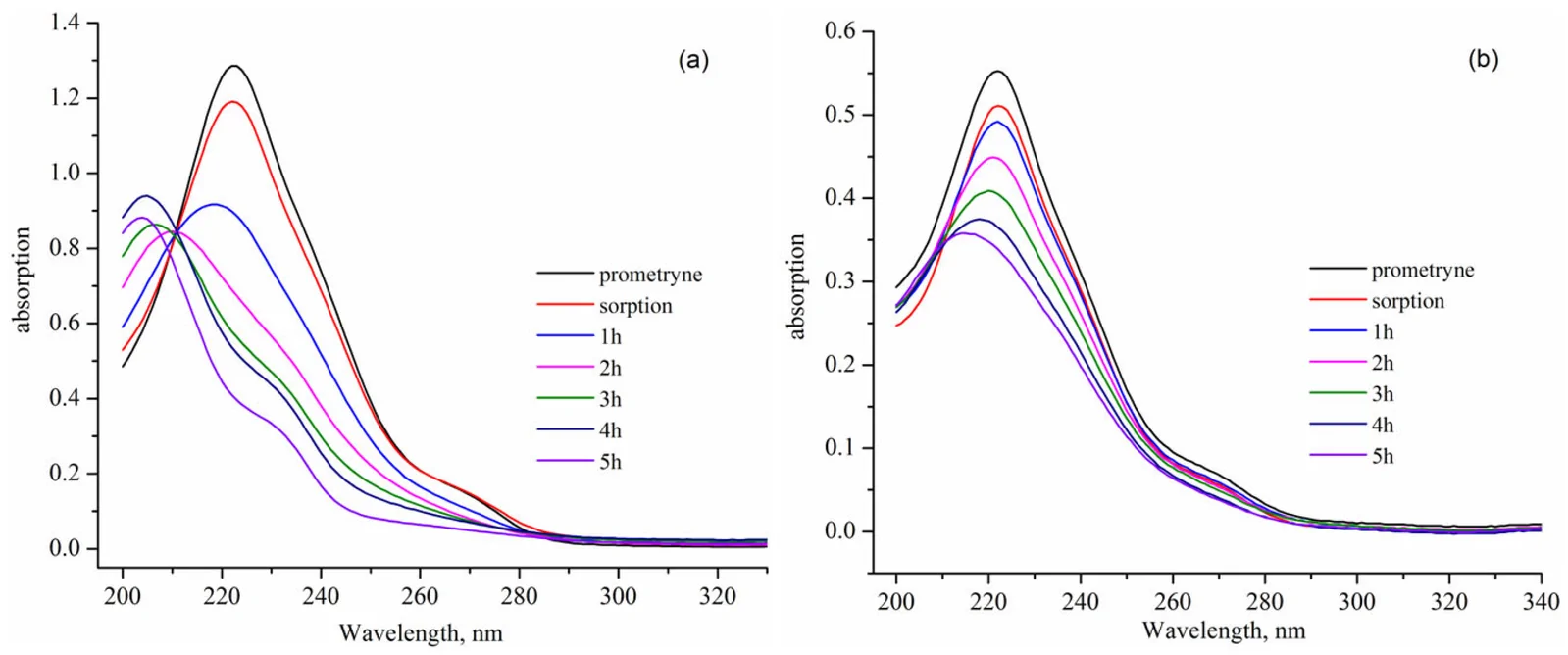
UV–vis absorption spectra of a PROM solution during 5 h of UV irradiation using ZnO(1)—(**a**) and ZnO(2) samples—(**b**).

UV–vis absorption spectra (presented on Figure 9) of prometryn show a decrease in absorption intensity (so-called “hypochromic effect”) at 221 nm wavelength, which corresponds to the maximum absorption peak for prometryn in water solution. Simultaneous shifting of the absorption peak in the “blue” region (hypsochromic shift) from 221 nm up to 205 nm occurred, which is more noticeable in ZnO(1) sample (Figure 9a). The above indicates the mineralization of triazine herbicide up to dissolved organic carbon (DOC), SO_4_^2−^, NO_3_^−^, and NH_4_^+^.

The photocatalytic degradation of prometryn was satisfactorily described by the pseudo-first-order kinetic model. For prometryn, the linearized pseudo-first-order plots gave correlation coefficients of R = 0.974 for ZnO(1) and R = 0.993 for ZnO(2). The apparent degradation rate constant of ZnO(2) was approximately two times higher than that of ZnO(1) (*K_d_* = 0.14 × 10^−2^ and 0.07 × 10^−2^ min^−1^, respectively; Table 4). Because no independent adsorption isotherms or intraparticle diffusion analysis were performed, the contribution of adsorption and pore diffusion processes cannot be quantified. It was found that synthesized samples are more effective in the CAP degradation process by 10 times for ZnO(1) and 4 times for ZnO(2) photocatalysts under UV irradiation than in PROM photodestruction reaction. Thus, it can be concluded that zinc oxide is a highly effective photocatalyst for destruction of emerging pollutants, including antibiotics (CAP) and herbicides (PROM). Also, the investigation of prometryn photodestruction was studied on TiO_2_/ZrO_2_ and BaO/ZrO_2_ compositions, which were synthesized by different methods and presented in [32,33,34].

Thus, it was established that ZnO powders are promising photocatalysts for the mineralization of medicines on the example of CAP and MNZ. For effective photocatalytic process ZnO must have a high specific surface area (to increase the efficiency of organic compounds adsorption), small particle sizes (for the probability of reducing the recombination of electrons and holes), as well as the ability to absorb photons from the visible part of the solar spectrum.

### 3.3. Catalytic Properties

The catalytic oxidation of ethanol over the synthesized nanostructures ZnO is also reported. Obtained results show that carbon oxides (CO_x_), hydrogen, acetaldehyde and ethylene are the reaction products.

**Figure 10:**
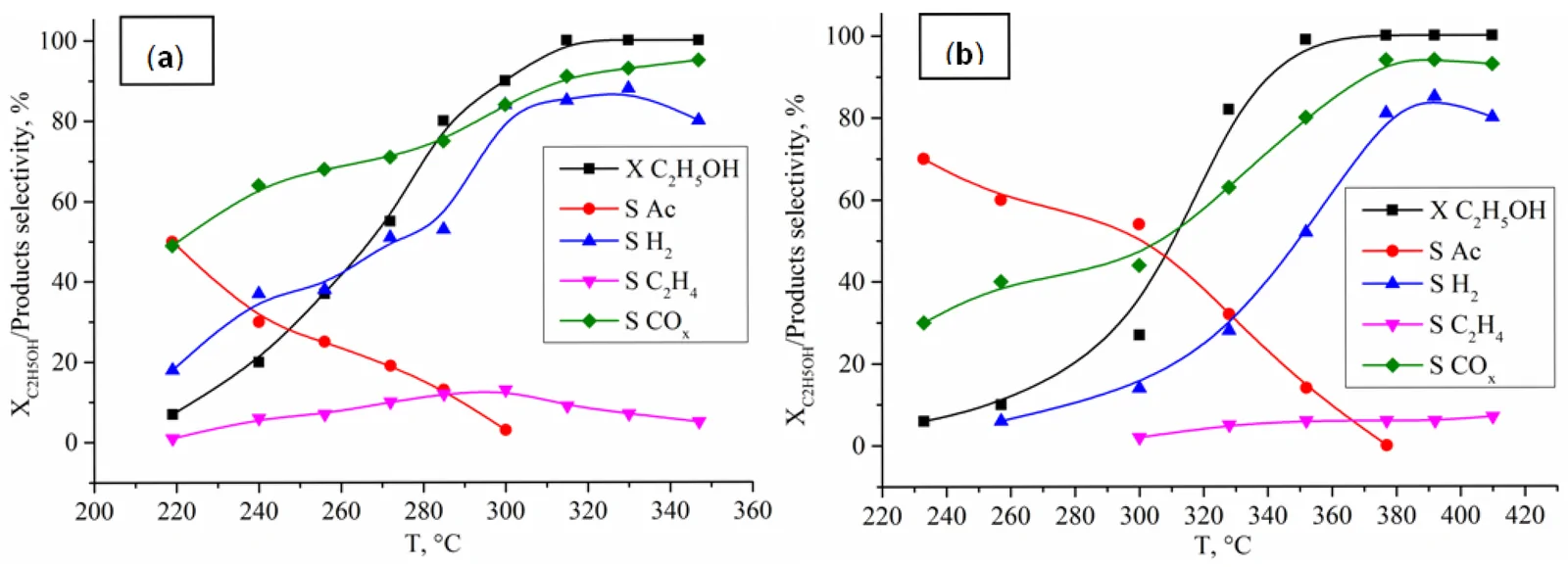
Temperature dependence of ethanol conversion and selectivity of reaction product formation on the samples: (**a**)—ZnO(1), (**b**)—ZnO(2); each catalytic experiment was repeated three times during heating and cooling cycles; the relative experimental error was 2–3%.

It was found that both zinc oxide catalysts have already a high activity at T ˃ 220 °C. As can be seen from Figure 10 both nanostructured catalysts show a sharp increase in alcohol conversion at 220–230 °C which is equal to 6–7%. At the same temperatures, the maximum selectivity for acetaldehyde is characteristic and is equal to 50% for ZnO(1) and 70% for ZnO (2) catalysts. At complete ethanol conversion, carbon oxides and hydrogen become the main reaction products, as described by Equation (12). Small amounts of ethylene are formed as a by-product through the parallel ethanol dehydration pathway described by Equation (13) [35,36,37].

*C*_2_*H*_5_*OH* + 1/2 *O*_2_ = *CH*_3_*CHO* + *H*_2_*O* (11)

*C*_2_*H*_5_*OH* + 1/2 *xO*_2_ = 2*COx* + 3*H*_2_ (12)

(13) $$C_{2}H_{5}\mathrm{OH}\overset{\;O_{2}\;}{\to }C_{2}H_{4}+H_{2}O$$

It is noteworthy that the selectivity of acetaldehyde formation sharply decreases with increasing reaction temperature for both samples and completely disappears at 315 °C and 377 °C for ZnO (1) and ZnO (2), respectively. Calculations of acetic aldehyde yield showed that the maximum value on ZnO (1) catalyst is 10.5% at 270 °C, while on ZnO (2) sample it is 26% at 328 °C. A similar percentage of acetic aldehyde was obtained in ethanol oxidation on the ZnO/MoO_3_ = 1/1 composites after mechanochemical milling [38] and sonochemical treatment [39]. The comparison of temperature dependence on ethanol conversion (Figure 10) for both oxides proves that the ZnO(1) is more active than ZnO(2). The catalytic behavior is also consistent with the observed differences in texture and morphology, although a direct causal assignment cannot be made from the available data. The higher surface area and total pore volume of ZnO(1), together with its less agglomerated morphology, may provide more accessible surface sites and contribute to its lower temperatures of 50% and complete ethanol conversion. In contrast, the porous agglomerated structure of ZnO(2) was associated with lower overall ethanol conversion activity but a higher maximum acetaldehyde selectivity. This result suggests that morphology and pore structure may affect the relative contribution of ethanol dehydrogenation and secondary reactions; however, detailed surface-chemistry studies would be required to establish responsible active sites and reaction pathways. This fact is evidenced by the results of full alcohol conversion (X = 100%): on ZnO(1) catalyst this value is achieved at T = 315 °C, while on the ZnO(2) sample it is reached 50 °C later (at 377 °C).

Simultaneously, upon reaching full ethanol conversion, carbon oxides and hydrogen are main products. However, small amounts of ethylene are formed as a by-product due to the collateral alcohol dehydration reaction. In this case, the maximum value of the selectivity of the ethylene formation on ZnO(1) sample is 13% at 300 °C, while on the ZnO(2) catalyst at the same temperature the dehydration process has just begun, and the selectivity of ethylene formation does not exceed 7% at 410 °C. According to the results obtained from Table 5 the lower activity and selectivity of ZnO(2) catalyst in this reaction than ZnO(1) can be seen. Comparison of the obtained data showed that the full ethanol conversion on ZnO(1) catalyst occurs 62 °C earlier (at 315 °C) than in the case of ZnO(2)—at 377 °C.

Calculated values of catalyst productivity show that obtained samples possess unique properties. So, the maximum productivity of the ZnO(1) and ZnO(2) catalysts on hydrogen is about 310 mol/h × kg_cat_ at 330 °C and 300 mol/h × kg_cat_ at 390 °C, respectively. It was found that increase in reaction temperature leads to decrease in catalyst productivity. Thus, the prospects of using zinc oxide as a catalyst on an industrial scale for the hydrogen production from ethanol can be concluded.

## 4. Conclusions

This study demonstrates that nanocrystalline zinc oxide of high purity can be synthesized from a real post-production electrolyte waste stream originating from the hydrometallurgical zinc industry. The resulting material exhibits crystallite size, structural properties, and purity (with only trace amounts of Fe, Mn, and Pb) that closely approach those of commercial ZnO. The waste-derived ZnO was proven to show competitive photocatalytic performance in the degradation of persistent organic contaminants (chloramphenicol, metronidazole, and prometryn), as well as favorable selectivity in ethanol oxidation, when compared with commercial reference material. ZnO synthesized from zinc production waste (ZnO(2)) exhibited competitive photocatalytic performance compared to commercial ZnO (ZnO(1)), achieving 79% CAP degradation (Kd = 0.53 × 10^−2^ min^−1^) and 72% MNZ degradation (Kd = 0.43 × 10^−2^ min^−1^) under UV light, while showing higher activity in PROM degradation (32% removal) due to its mesoporous structure. Although ZnO(2) had a slightly lower specific surface area (24.9 m^2^/g) and pore volume (0.26 cm^3^/g) than ZnO(1), it demonstrated significant environmental and economic benefits by repurposing industrial waste. Catalytic tests in ethanol oxidation revealed ZnO(2) required higher temperatures for full conversion but exhibited higher acetaldehyde selectivity (70%).

The results highlight the feasibility of producing advanced functional materials from industrial waste, thereby supporting the principles of the circular economy by reducing both raw material consumption and industrial waste generation. Importantly, the synthesis does not require extensive purification to reach impurity levels typical for commercial photocatalysts, showcasing its practicality and potential for scale-up.

Further research on optimizing the ZnO synthesis process from waste could contribute to improving its photocatalytic properties, bringing them closer to the parameters of commercial ZnO.

The method of synthesizing ZnO from waste electrolyte after zinc production can contribute to reducing industrial waste and alleviating pressure on natural resources. The application of as synthesized ZnO in photocatalytic water treatment may represent a step towards more sustainable and economical water purification methods, particularly in the context of removing difficult to eliminate contaminants. The present results were obtained under laboratory-scale conditions using model aqueous solutions. Further work should assess catalyst stability, reusability, transformation-product toxicity, and performance in real wastewater matrices. Pilot-scale studies will also be required to evaluate the operational feasibility of integrating waste-derived ZnO into practical photocatalytic water-treatment systems.

## Figures and Tables

**Table 1:** Chemical reaction equations corresponding to the four-stage synthesis process of ZnO from zinc-bearing industrial waste electrolyte, as schematically presented in Figure 1.

Stage	No.	Reaction
1. Precipitation	R1	ZnSO_4_ + H_2_C_2_O_4_·2H_2_O → ZnC_2_O_4_·2H_2_O + H_2_SO_4_
1. Precipitation	R2	MnSO_4_ + H_2_C_2_O_4_·2H_2_O → MnC_2_O_4_·2H_2_O + H_2_SO_4_
2. Ammonization	R3	ZnC_2_O_4_·2H_2_O + 4 NH_4_OH → Zn(NH_3_)_4_C_2_O_4_ + 6 H_2_O
2. Ammonization	R4	MnC_2_O_4_·2H_2_O + 4 NH_4_OH → Mn(NH_3_)_4_C_2_O_4_ + 6 H_2_O
Mn removal	R5	Mn(NH_3_)_4_C_2_O_4_ + 2 (NH_4_)_2_S_2_O_8_ + 4 NH_4_OH + H_2_O → 2 (NH_4_)_2_CO_3_ + 4 (NH_4_)_2_SO_4_ + MnO(OH)_2_
3. Deammonization/ reprecipitation	R6	Zn(NH_3_)_4_C_2_O_4_ + 2 H_2_SO_4_ + 2 H_2_O → ZnC_2_O_4_·2H_2_O + 2 (NH_4_)_2_SO_4_
3. Deammonization/ reprecipitation	R7	(NH_4_)_2_CO_3_ + H_2_SO_4_ → (NH_4_)_2_SO_4_ + CO_2_ + H_2_O
3. Deammonization/ reprecipitation	R8	2 NH_4_OH + H_2_SO_4_ → (NH_4_)_2_SO_4_ + 2 H_2_O
4a. Drying	R9	ZnC_2_O_4_·2H_2_O → ZnC_2_O_4_ + 2 H_2_O
4b. Calcination	R10	2 ZnC_2_O_4_ + O_2_ → 2 ZnO + 4 CO_2_

**Table 2:** Selected parameters of photocatalytic experiments.

Parameter	Case 1	Case 2	Case 3
Compound to be degraded	Chloramphenicol (CAP)	Metronidazole (MNZ)	Prometryn (PROM)
Formula	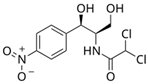	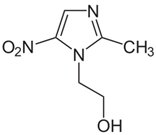	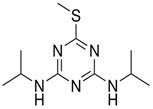
* λ_max_, nm	275	320	221
C, mol/L	7.50 × 10^−5^	8.76 × 10^−5^	1.38 × 10^−6^
pH	5.4	8.9	7.6

* λ_max_ is a characteristic absorption band of organic pollutant; C is an initial solution concentration for analysis.

**Table 3:** Elemental composition of waste-derived ZnO.

Element	Content (wt. %)
Zn	79.7
Fe	0.015
Mn	0.021
Na	0.015
Pb	0.009
Mg	0.006
K	0.002
Ca	0.045
Si	0.031
Cl	0.011

**Table 4:** Physicochemical and photocatalytic properties of ZnO samples.

***Parameter***	ZnO(1)		ZnO(2)	
* S_BET_, m^2^/g	27.4		24.9	
V_Ʃ_, cm^3^/g	0.52		0.26	
Kd × 10^2^, min^−1^	CAP			
	UV	Vis light (Na lamp)	UV	Na
	0.66	0.18	0.53	0.04
G, %	86	46	79	12
Kd × 10^2^, min^−1^	Prometryn			
	0.07	-	0.14	-
G, %	26	-	32	-
Kd × 10^2^, min^−1^	Metronidazole			
	UV	Visible light (Na lamp)	UV	Na
	1.12	-	0.43	0.01
G, %	96	-	72	2

* S_BET_ is a specific surface area of the sample by the BET method, V_Ʃ_ is a total pore volume, Kd is a degradation constant rate of chloramphenicol and prometryn solutions, and G is a removal degree of pollutant.

**Table 5:** Catalytic performance of ZnO samples in ethanol oxidation reaction.

Sample	T_r_, °C		Maximal Selectivity (at T_r_, °C)			
	* X_50%_	X_100%_	CO_x_	H_2_	C_2_H_4_	CH_3_CHO
ZnO(1)	267	315	95 (347)	88 (330)	13 (300)	50 (219)
ZnO(2)	311	377	94 (377)	85 (392)	7 (410)	70 (233)

* X—ethanol conversion, T_r_—temperature of reaction.

## Data Availability

The data presented in this study are available on request from the corresponding author.
